# Prenatal Bisphenol A Exposure Impairs Fetal Heart Development: Molecular and Structural Alterations with Sex-Specific Differences

**DOI:** 10.3390/antiox14070863

**Published:** 2025-07-14

**Authors:** Alessandro Marrone, Anna De Bartolo, Vittoria Rago, Francesco Conforti, Lidia Urlandini, Tommaso Angelone, Rosa Mazza, Maurizio Mandalà, Carmine Rocca

**Affiliations:** 1 Cellular and Molecular Cardiovascular Physiology and Pathophysiology Laboratory, Department of Biology, E. and E. S. (DiBEST), University of Calabria, 87036 Rende, Italy; alessandro.marrone@unical.it (A.M.); anna.de_bartolo@unical.it (A.D.B.); tommaso.angelone@unical.it (T.A.); 2 Human Anatomy Laboratory, Department of Pharmacy, Health and Nutritional Sciences, University of Calabria, 87036 Rende, Italy; vittoria.rago@unical.it (V.R.); lidiamaria.urlandini@unical.it (L.U.); 3 Pathology Unit, Annunziata Hospital, 87100 Cosenza, Italy; francesco.conforti@unical.it; 4 National Institute of Cardiovascular Research (INRC), 40196 Bologna, Italy; 5 System and Organ Physiology Laboratory, Department of Biology, E. and E. S. (DiBEST), University of Calabria, 87036 Rende, Italy; rosa.mazza@unical.it; 6 Vascular Physiology Laboratory, Department of Biology, E. and E. S. (DiBEST), University of Calabria, 87036 Rende, Italy

**Keywords:** bisphenol A, fetal heart development, endocrine disruption, heart disease, sex differences

## Abstract

Cardiovascular diseases (CVDs) remain the leading cause of morbidity and mortality worldwide, with increasing evidence suggesting that their origins may lie in prenatal life. Endocrine-disrupting chemicals (EDCs), such as bisphenol A (BPA), have been implicated in the alteration of fetal programming mechanisms that cause a predisposition to long-term cardiovascular vulnerability. However, the impact of prenatal endocrine disruption on fetal heart development and its sex-specific nature remains incompletely understood. This study investigates the molecular and structural effects of low-dose prenatal BPA exposure on fetal rat hearts. Our results reveal that BPA disrupts estrogen receptor (ER) signaling in a sex-dependent manner, with distinct alterations in ERα, ERβ, and GPER expression. BPA exposure also triggers significant inflammation, oxidative stress, and ferroptosis; this is evidenced by elevated NF-κB, IL-1β, TNF-α, and NLRP3 inflammasome activation, as well as impaired antioxidant defenses (SOD1, SOD2, CAT, and SELENOT), increased lipid peroxidation (MDA) and protein oxidation, decreased GPX4, and increased ACSL4 levels. These alterations are accompanied by increased markers of cardiac distension (ANP, BNP), extracellular matrix remodeling mediators, and pro-fibrotic regulators (Col1A1, Col3A1, TGF-β, and CTGF), with a more pronounced response in males. Histological analyses corroborated these molecular findings, revealing structural alterations as well as glycogen depletion in male fetal hearts, consistent with altered cardiac morphogenesis and metabolic stress. These effects were milder in females, reinforcing the notion of sex-specific vulnerability. Moreover, prenatal BPA exposure affected myocardial fiber architecture and vascular remodeling in a sex-dependent manner, as evidenced by reduced expression of desmin alongside increased levels of CD34 and Ki67. Overall, our findings provide novel insights into the crucial role of prenatal endocrine disruption during fetal heart development and its contribution to the early origins of CVD, underscoring the urgent need for targeted preventive strategies and further research into the functional impact of BPA-induced alterations on postnatal cardiac function and long-term disease susceptibility.

## 1. Introduction

Embryonic development is a highly regulated and dynamic process that plays a crucial role in determining long-term offspring health [1,2,3]. Disruptions in gene expression and cellular differentiation during critical windows of organogenesis, when the fetus is particularly vulnerable to external stimuli, may alter fetal programming, increasing the risk of congenital anomalies or predisposing individuals to chronic diseases in adulthood [4,5,6]. Among these disorders, cardiovascular diseases (CVDs)—the leading causes of morbidity and mortality worldwide—are of particular concern, as substantial evidence suggests that they may originate in utero [7,8,9,10]. Cardiac development depends on the precise timing and regulation of transcriptional activity in cardiomyoblasts; therefore, an adverse placental environment can disrupt gene expression, impairing proper heart formation [11,12].

Over the past decades, endocrine-disrupting compounds (EDCs) have gained increasing attention due to their interference with key signaling pathways involved in cardiomyocyte differentiation and homeostasis [13,14]. Among the various EDCs, bisphenol A (BPA) stands out as one of the most widely produced industrial chemicals. It is primarily used in the manufacture of polycarbonate plastics and epoxy resins, which are found in food and beverage containers as well as numerous daily consumer products. BPA-related health risks stem from its incomplete polymerization, leaving unbound monomers that leach into food and beverages, particularly under heat or acidic/basic conditions [15,16]. Consequently, dietary intake represents the primary route of human BPA exposure, although evidence indicates that inhalation, dermal absorption, and intravenous exposure also contribute [17].

As a result of continuous exposure, BPA has been extensively detected in human biological fluids, such as serum and urine. Moreover, it has also been found in umbilical cord blood, placenta, amniotic fluid, neonatal blood, colostrum, and breast milk, highlighting its potential for maternal–fetal transfer [18]. Unlike adults, who efficiently conjugate BPA into BPA-glucuronide (BPA-GA) in the liver for excretion via bile, the fetus has a limited metabolic capacity, leading to BPA-GA deglucuronidation and increased exposure to its biologically active form [19,20]. Although its exact mechanisms of action remain incompletely understood, BPA exerts its biological effects through complex genomic and non-genomic pathways involving various intracellular hormone receptors. However, as a xenoestrogen, BPA primarily targets the classical nuclear estrogen receptors ERα and ERβ, as well as the membrane-bound G protein-coupled estrogen receptor 30 (GPR30, also known as GPER) [21]. While these receptors play a significant role in mediating BPA’s effects, whether BPA activates or inhibits the associated signaling cascades remains unclear.

Despite extensive in vitro and in vivo evidence linking BPA exposure to adverse cardiovascular effects [22,23], its impact on fetal heart development remains largely unexplored. The need for a deeper understanding of BPA’s mechanisms of action is highlighted by recent findings suggesting that BPA acts as a selective estrogen receptor modulator (SERM) [24]. This implies that receptor-mediated responses to BPA can vary depending on the dose, duration, and timing of exposure, as well as age, sex, and the specific tissue and cell types involved [24].

Given these considerations, this study aims to investigate the molecular mechanisms underlying the effects of prenatal BPA exposure on fetal heart development. Using a rat model, we examined key pathways involved in cardiac remodeling and fibrosis, assessing both structural and molecular alterations. Considering the pivotal role of estrogenic signaling in cardiac development, we also explored sex-specific differences in BPA-induced effects, particularly regarding estrogen receptor regulation. Notably, the dose used in this study (2.5 µg/kg/day) aligns with levels of human exposure reported in epidemiological studies [25], underscoring the gap between real-world exposure and current safety thresholds. This reinforces concerns about the potential cardiovascular risks associated with low-dose BPA exposure. Elucidating these mechanisms could provide critical insights into the developmental origins of cardiovascular pathologies and support ongoing efforts to refine BPA safety guidelines.

## 2. Materials and Methods

### 2.1. Animals and Treatments

All experiments were conducted on fetuses isolated from pregnant Sprague–Dawley rats. The animals were randomly assigned to two experimental groups: one receiving bisphenol A (BPA) at a dose of 2.5 μg/kg/day dissolved in ethanol (n = 4), while the control group received ethanol (Et-OH) alone, the vehicle used for BPA administration (n = 4). BPA or Et-OH was administered via drinking water for one month prior to conception and throughout the 21 days of gestation. All rats were individually housed in the Animal Care Facility under controlled environmental conditions (12-h light/dark cycle), with free access to commercial chow and tap water. At the end of the experimental period, dams were euthanized by isoflurane inhalation followed by decapitation using a small-animal guillotine. Fetuses were promptly collected and sexed based on the anogenital distance (AGD), measured under a stereomicroscope. Fetal hearts were then carefully isolated and either processed for histological analysis or stored at −80 °C for subsequent protein extraction (used for Western blotting and gelatin zymography, and biochemical analyses) and RNA isolation (used for quantitative real-time PCR, qPCR). The analyses were performed on the pooled fetal heart samples (n = 4 per pregnant rat/group, with a total of n = 4 pregnant rats/group) across n = 3 independent experiments. All experimental procedures were conducted in accordance with the European Guidelines for the Care and Use of Laboratory Animals (Directive 2010/63/EU) and were approved by the local ethical committee of the University of Calabria and the Italian Ministry of Health (authorization n. 104/2024-PR).

### 2.2. Western Blot Analysis

Fetal rat hearts from each experimental group were pooled (n = 4 per group) and homogenized in ice-cold RIPA lysis buffer supplemented with a phosphatase and protease inhibitor cocktail and centrifuged at 14,000 rpm for 20 min at 4 °C, as previously described [26,27]. The resulting supernatant was collected, and the protein concentration was determined using the Bradford assay. Equal amounts of protein (40 μg) were separated on SDS-PAGE gels (with different acrylamide concentrations) of different concentrations, depending on the target protein: 8% SDS-PAGE for NLRP3; 10% SDS-PAGE for ERα, ERβ, GPER, CTGF, CAT, SELENOT, ACSL4, and NF-κB p65; 12% SDS-PAGE for SOD1, SOD2, and GPX4. Proteins were then transferred onto nitrocellulose membranes using the Trans-Blot Turbo system (Bio-Rad, Hercules, CA, USA). Membranes were blocked with 5% non-fat dried milk at room temperature for 1 h, washed three times with Tris-buffered saline containing 0.1% Tween 20 (TBST), and incubated overnight at 4 °C with specific primary antibodies diluted in TBST containing either 5% bovine serum albumin (BSA) or 1% non-fat dried milk, as follows: ERα (1:500, sc-8002), Erβ (1:500, sc-8974), GPER1 (1:500, ab-137479), NLRP3/NALP3 (1:300, NBP2-12446), SOD1 (1:1000, sc-101523), SOD2 (1:1000, sc-133134), CATALASE (1:1000, sc-271803), SELENOT (1:500, LS-C168948), CTGF (1:500, TA806803), GPX4 (1:1000, NBP2-75511), ACSL4 (1:1000, 22401-1-AP), and NF-κB p65 (1:500, sc-8008). The β-actin antibody (1:1000, SC-47778 C4) was used as the loading control. After washing, membranes were incubated with peroxidase-conjugated secondary antibodies at room temperature for 1 h. The following dilutions were used: Anti-rabbit (1:2000) and Anti-mouse (1:1000). Immunodetection was performed using the Clarity Western ECL Substrate (Bio-Rad). Band densitometry analysis was carried out by measuring the area and pixel intensity using ImageJ 1.6 software (National Institutes of Health, Bethesda, MD, USA), as previously described [28].

### 2.3. Gelatin Zymography for Matrix Metalloproteinase Activity Detection

The enzymatic activity of matrix metalloproteinases (MMPs) was assessed by SDS-PAGE zymography using gelatin as a substrate, as previously reported [29]. Briefly, equal amounts of protein (60 μg) from fetal heart samples were loaded under non-denaturing conditions onto 8% SDS-PAGE gels containing 0.1% gelatin. After electrophoresis, gels were washed for 30 min in Buffer I [50 mM Tris-HCl (pH 7.5), 10 mM CaCl_2_, 2.5% Triton X-100] to remove SDS and allow enzyme renaturation. Gels were then incubated overnight at 37 °C in Buffer II [50 mM Tris–HCl (pH 7.5), 150 mM NaCl, 5 mM CaCl_2_] to enable enzymatic activity. Finally, gels were stained with 2% Coomassie Brilliant Blue R-250 (Sigma Aldrich, St. Louis, MO, USA), 25% methanol, and 10% acetic acid, followed by destaining in 2% methanol and 4% acetic acid until clear bands appeared. Gelatin digestion areas, indicative of MMP activity, were visualized and quantified using ImageJ 1.6 software. MMP-2 activity was expressed as a percentage of the total band area across the four experimental groups.

### 2.4. Gene Expression Analysis by qPCR

Total RNA was isolated from pooled fetal hearts (n = 4 per group) using TRIzol™ reagent (Invitrogen, Waltham, MA, USA) following the manufacturer’s protocol. 2 μg of total RNA were reverse-transcribed into cDNA using the High-Capacity cDNA Reverse Transcription Kit (Applied Biosystems™, Thermo Fisher Scientific, Waltham, MA, USA). qPCR amplification was performed using SYBR™ Green Master Mix (Thermo Fisher Scientific) according to the manufacturer’s instructions on a QuantStudio™ 1 Real-Time PCR System apparatus (Thermo Fisher Scientific). Each sample was analyzed in duplicate, with three independent experiments (n = 3). 18S rRNA was used as an internal control, and primer sequences used for amplification are listed in Table 1. Relative gene expression levels were calculated using the 2^−ΔΔCt^ method [30].

### 2.5. Evaluation of Malondialdehyde (MDA) Levels

Lipid peroxidation in fetal heart tissues was determined by measuring thiobarbituric acid reactive substances (TBARS). Briefly, pooled fetal hearts (n = 4) from each group were homogenized in 0.9% KCl solution (pH 7.4) at 10% *w*/*v*. Samples were processed following protocols described in previous publications [31,32]. TBARS levels were spectrophotometrically detected at 523 nm and expressed as nmol/g heart tissue.

### 2.6. Detection of Protein Carbonylation

Protein oxidation in fetal hearts was assessed using the 2,4-dinitrophenylhydrazine (DNPH) method [33]. Cardiac tissues were homogenized in 50 mM phosphate buffer (pH 7.4) containing 1% dithiothreitol (DTT) and a protease inhibitor cocktail (1:100 *v*/*v*) (Sigma Aldrich). The total protein concentration in the cardiac extracts was determined by the Bradford assay. The procedure was carried out according to previously described protocols [34,35,36]. Protein carbonyl content was measured spectrophotometrically at 375 nm using a Multiskan™ SkyHigh spectrophotometer (Thermo Fisher Scientific Inc.), against the relative control absorbances using an extinction coefficient of 22,000 M^−1^ cm^−1^. The results were expressed as nmol carbonyl groups/mg protein.

### 2.7. Histological Analysis

Fetal heart tissue from rats exposed to BPA or the vehicle was fixed in 4% paraformaldehyde (PFA), embedded in paraffin, and sectioned at 5 μm thickness. The deparaffinized and rehydrated sections were stained with hematoxylin and eosin (H&E), Masson’s trichrome (04-010802, Bio Optica, Milano, Italy), and Schiff’s periodic acid (PAS, 04-130802, Bio Optica), using standard procedures and according to the manufacturer’s instructions. Image analysis of the cardiac tissue was obtained with an Olympus BX41 fluorescence microscope and acquired with CSV1.14 software, using the CAMXC-30 camera (Olympus, Shinjuku, Tokyo, Japan) for acquisition.

### 2.8. Immunofluorescence and Immunohistochemistry

Immunofluorescence and immunohistochemical analyses of fetal cardiac sections were conducted on deparaffinized and rehydrated serial sections. Briefly, sections of fetal heart tissue were rinsed with DPBS, permeabilized with 0.1% Triton X-100 in DPBS for 30 min at RT, and subsequently blocked with 1% BSA in DPBS for 30 min at RT. Following these steps, the sections were incubated overnight at 4 °C with the anti-Desmin primary antibody (PA5-16705, Thermo Fisher Scientific), diluted 1:100. Thereafter, the sections were incubated with a goat anti-rabbit secondary antibody—Alexa Fluor™ 488 (diluted 1:1200) for 1 h at RT, in accordance with the manufacturer’s instructions (Invitrogen). Sections were washed three times with DPBS, and nuclei were counterstained with DAPI. Fluorescence images were acquired using an Olympus BX41 fluorescence microscope (Olympus) with CSV1.14 software and a CAMXC-30 camera for acquisition. Fluorescence quantification was performed using ImageJ 1.6 software (National Institutes of Health, Bethesda, MD, USA).

For immunohistochemical staining, following heat-mediated antigen retrieval, cardiac sections were incubated overnight at 4 °C with primary antibodies diluted 1:100 against CD34 (AF4117, Novus Biologicals, Centennial, CO, USA) and Ki67 (AB9260, Sigma Aldrich). Then, biotinylated IgG (1:600) was applied for 1 h at RT, followed by avidin-biotin complex (ABC)/horseradish peroxidase (HRP). Immunoreactivity was detected using diaminobenzidine tetrahydrochloride (DAB), followed by nuclear counterstaining with hematoxylin. Images were captured using an Olympus BX41 fluorescence microscope with CSV1.14 software and a CAMXC-30 camera. CD34 positivity of vessels was assessed by two independent, blinded observers through examination of the entire section area, and the results were expressed as the number of CD34-positive vessels.

### 2.9. Statistical Analysis

Data are expressed as the mean ± SEM. Statistical analyses were performed using two-way ANOVA, followed by the nonparametric Newman–Keuls multiple comparison test (for post hoc ANOVA comparisons). Statistical significance was set at *p* < 0.05 (*), *p* < 0.01 (**), *p* < 0.001 (***), and *p* < 0.0001 (****). All analyses were conducted using Prism 5 (GraphPad Software, La Jolla, CA, USA).

## 3. Results

### 3.1. Prenatal BPA Exposure Alters Estrogen Receptor Expression in a Sex-Specific Manner in the Fetal Rat Heart

**Figure 1:**
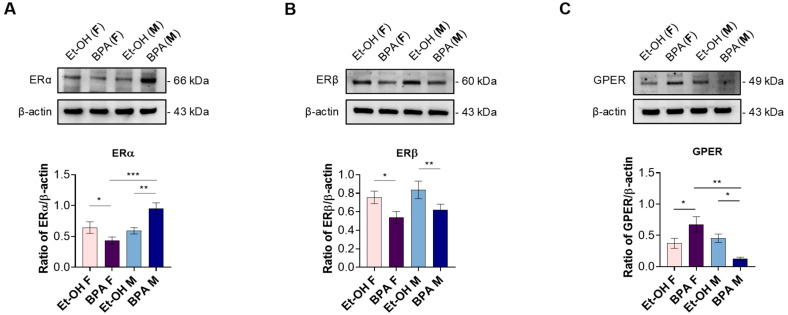
Effect of BPA on estrogen receptors (ER) and G protein-coupled estrogen receptor (GPER) in cardiac tissues. (**A**) Western blot analysis of ERα in the hearts of Et-OH female, BPA female, Et-OH male, and BPA male groups. Significant differences were detected by two-way ANOVA and Newman–Keuls multiple comparison test. * *p* < 0.05; ** *p* < 0.01; *** *p* < 0.001. (**B**) Western blot analysis of ERβ in the hearts of Et-OH female, BPA female, Et-OH male, and BPA male groups. Significant differences were detected by two-way ANOVA and Newman–Keuls multiple comparison test. * *p* < 0.05; ** *p* < 0.01. (**C**) Western blot analysis of GPER in the hearts of Et-OH female, BPA female, Et-OH male, and BPA male groups (n = 4 pulled). Histograms represent the ratio of densitometric analysis of protein:loading control. Data are expressed as the mean ± SEM (n = 3 independent experiments). Significant differences were detected by two-way ANOVA and Newman–Keuls multiple comparison test. * *p* < 0.05; ** *p* < 0.01.

To gain initial insight into the potential mechanism of action of BPA in male and female fetal rat hearts, we first assessed its effects on the protein expression levels of the classical nuclear estrogen receptors ERα and ERβ, as well as the orphan membrane-bound G protein-coupled estrogen receptor 30 (GPR30, also known as GPER). Figure 1A shows that, at baseline, males and females exhibited comparable ERα levels; however, females showed a significant decrease in the fetal heart levels of ERα compared to vehicle-treated controls, whereas males showed a significant increase compared to the ET-OH-treated group. Moreover, ERα expression was significantly higher in males than females (Figure 1A).

Regarding fetal cardiac ERβ expression, prenatal BPA exposure induced a significant reduction in both sexes compared to the control (Figure 1B).

The assessment of fetal cardiac GPER expression revealed that females exhibited a significant increase in receptor expression compared to the control, whereas males showed a significant downregulation. This differential modulation resulted in a pronounced sex difference in GPER expression following prenatal BPA exposure (Figure 1C).

### 3.2. Prenatal BPA Exposure Triggers Inflammation, Oxidative Stress, and Ferroptosis in the Fetal Rat Heart

**Figure 2:**
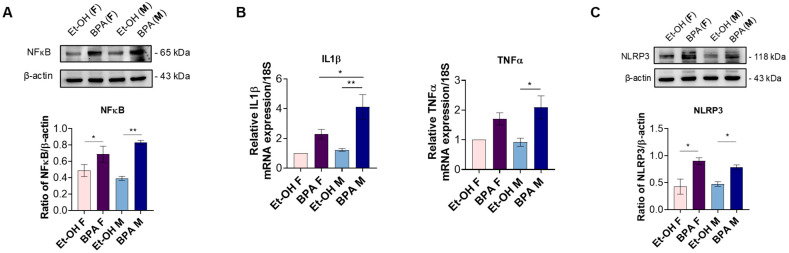
Effects of BPA on embryonic cardiac tissues. (**A**) Western blot analysis of NFkB in the hearts of Et-OH female, BPA female, Et-OH male, and BPA male groups (n = 4 pulled). Histograms represent the ratio of densitometric analysis of protein:loading control. Data are expressed as the mean ± SEM (n = 3 independent experiments). Significant differences were detected by two-way ANOVA and Newman–Keuls multiple comparison test. * *p* < 0.05; ** *p* < 0.01. (**B**) Evaluation of mRNA expression levels of IL1 β and TNFα in Et-OH female, BPA female, Et-OH male, and BPA male groups (n = 4 pulled). The relative mRNA expression levels of pro-inflammatory genes were normalized to 18S rRNA. Fold change is calculated on the basis of the 2^−ΔΔCT^. Significant differences were detected by two-way ANOVA and Newman–Keuls multiple comparison test. * *p* < 0.05; ** *p* < 0.01. (**C**) Western blot analysis of NLRP3 in the hearts of Et-OH female, BPA female, Et-OH male, and BPA male groups (n = 4 pulled). Histograms represent the ratio of densitometric analysis of protein:loading control. Data are expressed as the mean ± SEM (n = 3 independent experiments). Significant differences were detected by two-way ANOVA and Newman–Keuls multiple comparison test. * *p* < 0.05.

To determine whether alterations in estrogen receptors might be linked to inflammatory signaling, we assessed the expression of NF-κB, a key redox- and hormone-sensitive transcription factor involved in immune and stress responses [37]. Western blot analysis revealed a significant increase in NF-κB protein levels in BPA-exposed fetal hearts compared to controls, with a more pronounced effect in males (even if in a non-significant manner) (Figure 2A).

We next investigated whether prenatal BPA exposure leads to the upregulation of downstream inflammatory mediators. We assessed the gene expression levels of IL-1β and TNF-α, two key pro-inflammatory cytokines involved in cardiac dysfunction [38]. qPCR analysis revealed a significant upregulation of both markers in BPA-exposed male fetuses compared to the control. In female fetuses, although a similar trend was observed, the difference did not reach statistical significance. Moreover, in BPA-exposed fetuses, IL-1β expression was significantly higher in males than in females (Figure 2B).

Given the role of the NLRP3 inflammasome multiprotein complex in amplifying inflammatory signaling and mediating IL-1β activation [39], we further assessed its protein expression levels through Western blot and relative densitometric analyses. Our results revealed a significant increase in NLRP3 expression in both sexes prenatally exposed to BPA compared to the control (Figure 2C).

**Figure 3:**
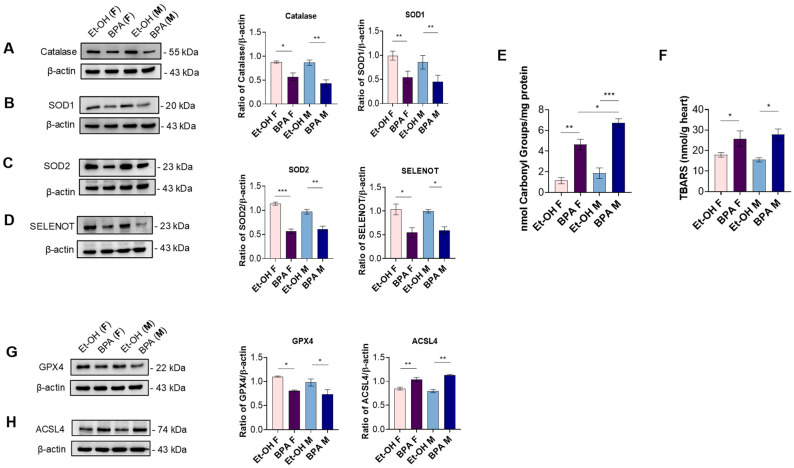
Effect of BPA on indicators of oxidative stress in cardiac tissues. (**A**) Western blot analysis of catalase in the hearts of Et-OH female, BPA female, Et-OH male, and BPA male groups (n = 4 pulled). Significant differences were detected by two-way ANOVA and Newman–Keuls multiple comparison test. * *p* < 0.05; ** *p* < 0.01 (**B**,**C**) Western blot analysis of SOD1 and SOD2 in the hearts of Et-OH female, BPA female, Et-OH male, and BPA male groups (n = 4 pulled). Significant differences were detected by two-way ANOVA and Newman–Keuls multiple comparison test. ** *p* < 0.01; *** *p* < 0.001. (**D**) Western blot analysis of SELENOT in the hearts of Et-OH female, BPA female, Et-OH male, and BPA male groups (n = 4 pulled). Histograms represent the ratio of densitometric analysis of protein:loading control. Significant differences were detected by two-way ANOVA and Newman–Keuls multiple comparison test. * *p* < 0.05. (**E**) Protein carbonyl groups in the hearts of Et-OH female, BPA female, Et-OH male, and BPA male groups (n = 4 pulled). Significant differences were detected by two-way ANOVA and Newman–Keuls multiple comparison test. * *p* < 0.05; ** *p* < 0.01; *** *p* < 0.001. (**F**) TBARS levels in the hearts of Et-OH female, BPA female, Et-OH male, and BPA male groups (n = 4 pulled). Significant differences were detected by two-way ANOVA and Newman–Keuls multiple comparison test. * *p* < 0.05. (**G**,**H**) Western blot analysis of GPX4 and ACSL4 in the hearts of Et-OH female, BPA female, Et-OH male, and BPA male groups (n = 4 pulled). Histograms represent the ratio of densitometric analysis of protein:loading control. Data are expressed as the mean ± SEM (n = 3 independent experiments). Significant differences were detected by two-way ANOVA and Newman–Keuls multiple comparison test. * *p* < 0.05; ** *p* < 0.01.

We next investigated the protein expression levels of key antioxidant enzymes to determine whether prenatal BPA exposure disrupts the antioxidant defense system in fetal rat hearts. Specifically, we assessed CAT, SOD1, and SOD2. As shown in the figure, prenatal BPA exposure led to a significant reduction in the expression levels of all three enzymes in both sexes compared to the control (Figure 3A–C).

Considering the importance of selenoproteins in redox balance and cellular protection, we further investigated the expression of selenoprotein T (SELENOT). Notably, BPA exposure significantly downregulated SELENOT expression in both sexes, compared to the control (Figure 3D).

To quantitatively assess the extent of oxidative stress in fetal cardiac tissue, we measured protein oxidation and lipid peroxidation through the DNPH method and TBARS assay, respectively. Prenatal BPA exposure significantly augmented protein carbonyl groups in male and female fetal hearts compared to their controls, with a more pronounced effect observed in males (Figure 3E). Similarly, BPA significantly increased TBARS levels in both sexes compared to their respective controls (Figure 3F).

Given the observed oxidative stress and lipid peroxidation, we investigated whether ferroptosis could contribute to the cardiac alterations induced by prenatal BPA exposure. Western blot analysis revealed a significant downregulation of glutathione peroxidase 4 (GPX4), a key enzyme protecting against ferroptosis by reducing lipid hydroperoxides [40]. Conversely, the expression of acyl-CoA synthetase long-chain family member 4 (ACSL4), which promotes the incorporation of polyunsaturated fatty acids into membrane phospholipids and sensitizes cells to ferroptosis [41], was significantly upregulated in BPA-exposed fetal hearts (Figure 3G,H).

### 3.3. Prenatal BPA Exposure Affects Markers of Cardiac Distension, Remodeling, and Fibrosis in the Fetal Rat Heart

**Figure 4:**
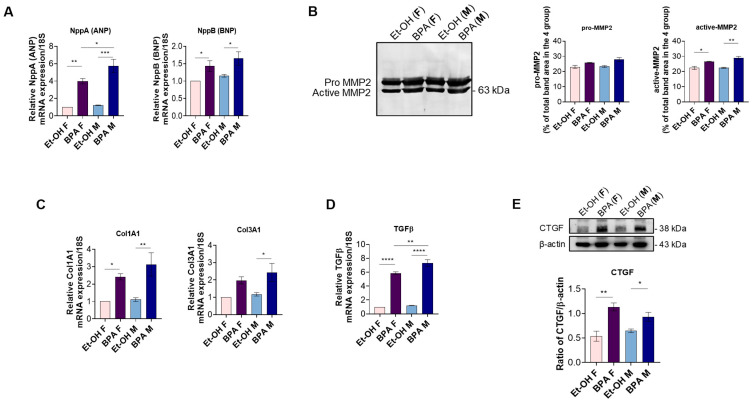
Effect of BPA on indicators of cardiac distension, remodeling, and fibrosis. (**A**) Evaluation of mRNA expression levels of *NppA* (ANP) and *NppB* (BNP) in Et-OH female, BPA female, Et-OH male, and BPA male groups (n = 4 pulled). Significant differences were detected by two-way ANOVA and Newman–Keuls multiple comparison test. * *p* < 0.05; ** *p* < 0.01; *** *p* < 0.001. (**B**) Gelatin SDS-PAGE zymographic analysis of MMP-2 activity in embryonic cardiac tissues in the hearts of Et-OH female, BPA female, Et-OH male, and BPA male groups (n = 4 pulled). Significant differences were detected by two-way ANOVA and Newman–Keuls multiple comparison test. * *p* < 0.05; ** *p* < 0.01 (**C**) Evaluation of mRNA expression levels of *Col1A1* and *Col3A1* in the hearts of Et-OH female, BPA female, Et-OH male, and BPA male groups (n = 4 pulled) Significant differences were detected by two-way ANOVA and Newman–Keuls multiple comparison test. * *p* < 0.05; ** *p* < 0.01 (**D**) mRNA expression levels of *TGFβ* in the hearts of Et-OH female, BPA female, Et-OH male, and BPA male groups (n = 4 pulled). Significant differences were detected by two-way ANOVA and Newman–Keuls multiple comparison test. ** *p* < 0.01; **** *p* < 0.0001. (**E**) Western blot analysis of CTGF in the hearts of Et-OH female, BPA female, Et-OH male, and BPA male groups (n = 4 pulled). Data are expressed as the mean ± SEM (n = 3 independent experiments). The relative mRNA expression levels were normalized to 18S rRNA. The fold change is calculated on the basis of 2^−ΔΔCT^. Histograms represent the ratio of densitometric analysis of protein:loading control. Significant differences were detected by two-way ANOVA and Newman–Keuls multiple comparison test. * *p* < 0.05; ** *p* < 0.01.

Given the role of inflammation and oxidative stress in triggering cardiac remodeling [42,43], we next evaluated whether prenatal BPA exposure affects markers of cardiac distension, remodeling, and fibrosis, thus promoting tissue alterations in fetal rat hearts. To this aim, we first assessed the gene expression levels of ANP and BNP. Both genes were significantly upregulated in the fetal hearts of BPA-exposed males and females compared to the control. Additionally, ANP expression was markedly higher in males than in females (Figure 4A).

We next assessed MMP2 gelatinolytic activity through zymography. Our results showed a significant upregulation of the active form of MMP2 (63 kDa) in both sexes prenatally exposed to BPA compared to the control. However, no significant differences were observed in its proenzyme form (66 kDa), indicating that BPA exposure may primarily affect MMP2 activation rather than its total expression (Figure 4B).

Additionally, we evaluated the gene expression levels of Col1A1 and Col3A1, two key markers of fibrotic progression and ECM deposition [44]. qPCR analysis revealed a significant increase in Col1A1 expression in the hearts of fetal rats prenatally exposed to BPA, in both sexes, compared to the control. Conversely, Col3A1 expression was significantly upregulated only in males, while females exhibited a similar trend that did not reach statistical significance (Figure 4C).

To gain deeper insight into the molecular mechanisms driving ECM reorganization following prenatal BPA exposure, we analyzed additional fibrosis-related markers. Specifically, through qPCR, we assessed TGF-β gene expression, a key regulator of fibrosis and ECM remodeling. As shown in the figure, TGF-β was significantly upregulated in the fetal hearts of both sexes prenatally exposed to BPA compared to the control. Moreover, a significant difference was observed between BPA-exposed males and females, suggesting a potential sex-specific regulation of pro-fibrotic signaling (Figure 4D).

On the other hand, we evaluated the expression of CTGF, a key downstream effector of TGF-β signaling and a critical driver of fibroblast activation, collagen deposition, and cardiac fibrosis [45]. Western blot analysis revealed a significant increase in CTGF protein levels in both sexes following BPA exposure compared to the control, further supporting the role of BPA in promoting a pro-fibrotic cardiac environment during fetal development (Figure 4E).

### 3.4. Prenatal BPA Exposure Induces Histological Alterations in the Fetal Rat Heart

**Figure 5:**
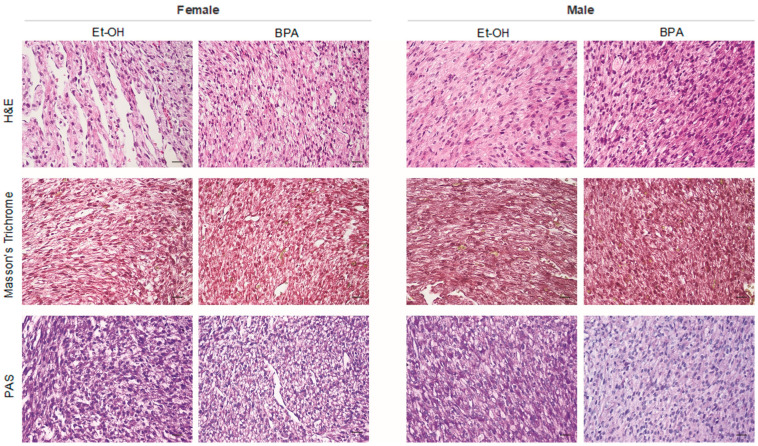
Morphological analysis was performed on embryonic hearts from male and female fetuses exposed to BPA or to the vehicle alone, Et-OH. The image is representative of n = 3 samples. Scale bars: 25 µm.

To determine whether prenatal BPA exposure induces structural and histopathological alterations in the fetal rat heart, we performed morphological analyses using H.E., Masson’s trichrome, and PAS staining (Figure 5).

The results obtained by H.E. showed that in the hearts of male control rats, the presence of cardiac fibers was not completely structured, an indication of incomplete tissue differentiation, while in the hearts of rats treated with BPA, H.E. staining showed an increase in cellularity, an indication of evident tissue hyperplasia, a marked anisotropy with widespread changes in spatial orientation of the fibers, and an increase in vessels not associated with an accompanying interstitial inflammatory infiltrate. No foci of necrosis were highlighted in the controls, while phases of tissue degeneration similar to necrobiosis were highlighted in the treated ones. In both samples, no variations in the mitotic index were highlighted. Similarly, in female control rats, H.E. staining revealed incomplete tissue differentiation, as observed in male controls. However, in the hearts of female rats treated with BPA, it highlighted a slight increase in cellularity, accompanied by a slight structural alteration of the fibers accompanied by a slight increase in vessels. No evident necrotic foci or changes in the mitotic index were detected in either group.

Masson’s trichrome staining showed the absence of tissue fibrosis in both controls and treated male and female samples.

Furthermore, PAS staining revealed a significant reduction in tissue glycogen levels in the hearts of male rats exposed to BPA compared to controls. In contrast, female hearts exhibited only a slight reduction in tissue glycogen levels between the control and BPA-treated groups.

**Figure 6:**
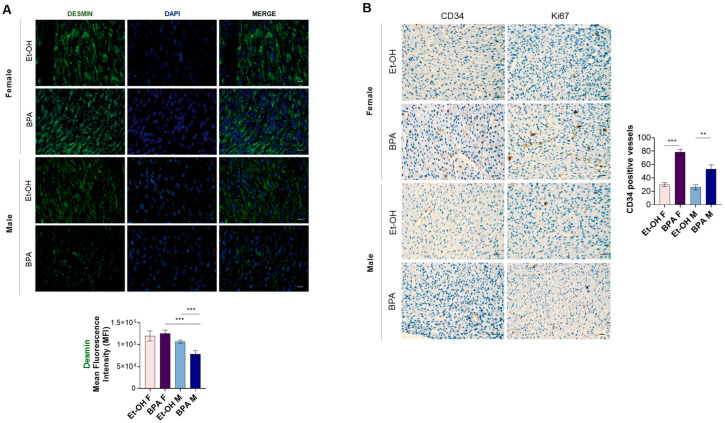
Effects of BPA on cardiac architecture. (**A**) Representative images of desmin levels and relative quantification in embryonic hearts from male and female fetuses exposed to BPA or to the vehicle, Et-OH. Desmin cardiac expression was assessed using an anti-desmin primary antibody and a goat anti-rabbit secondary antibody—Alexa Fluor™ 488 (green). Nuclei were counterstained with DAPI (blue). Scale bars: 12.5 µm. Significant differences were detected by two-way ANOVA and Newman–Keuls multiple comparison test. *** *p* < 0.001. (**B**) Immunohistochemical expression of CD34 with quantification of CD34 positive vessels (left panel) and immunohistochemical expression of Ki67 (right panel) in embryonic hearts from male and female fetuses exposed to BPA or to the vehicle, Et-OH. Scale bars: 25 µm. Significant differences were detected by two-way ANOVA and Newman–Keuls multiple comparison test. ** *p* < 0.01; *** *p* < 0.001.

To further investigate the structural damage induced by BPA exposure, we first assessed the expression of desmin, a major component of the muscle intermediate filaments, playing a vital role in the maintenance of skeletal and cardiac cellular architecture and structure [46] in fetal cardiac tissues. The results showed a clear localization of desmin at the Z-discs of the sarcomeres in both male and female control hearts, while they highlighted a reduced expression of desmin, especially in the cardiac fibers of male hearts exposed to BPA (Figure 6A). Moreover, to evaluate potential vascular damage associated with BPA exposure, we analyzed the expression of CD34, a cell surface antigen expressed on endothelial cells, commonly used as a marker to identify and quantify blood vessels in altered tissues [47]. As shown in Figure 6B, CD34 expression was increased in the microvessels of both male and female hearts from the BPA-treated groups compared to their controls, with a more pronounced effect observed in females. Based on the BPA-induced increase in cellularity and vessels observed in morphological evaluation, we then evaluated the expression of Ki67, a marker of cellular proliferation [48]. The results showed that the same tissue foci reactive for CD34 were reactive for Ki67, particularly in the female hearts treated with BPA (Figure 6B).

## 4. Discussion

Cardiovascular diseases remain the foremost cause of mortality worldwide, posing a critical challenge to public health systems and highlighting the urgent need for innovative prevention and treatment strategies [49]. Growing evidence supports the concept that these diseases may have their roots during embryonic development, a sensitive time-window during which genetic predispositions and environmental factors converge to shape the future cardiovascular health [50,51]. Understanding CVD etiology is thus paramount for shifting the focus toward triggering risk factors and enabling the implementation of population-wide early prevention strategies, rather than solely treating established conditions at the individual level.

Among the various environmental factors implicated in early cardiovascular programming, endocrine-disrupting chemicals, such as BPA, have gained increasing attention due to their widespread exposure and potential impact on fetal heart development. Numerous studies have investigated the cardiovascular effects of BPA, but many suffer from limitations related to model selection, exposure paradigms, or dose relevance. For instance, Chapalamadugu et al. (2014) [52] analyzed fetal heart transcriptomic alterations following BPA exposure in rhesus monkeys but used a very high dose (400 mg/kg/day), far exceeding realistic human exposure levels, potentially leading to non-physiological effects. Similarly, Guo et al. (2025) [53] demonstrated that high-dose BPA exposure induces congenital heart defects through mitochondrial dysfunction but did not assess whether these effects persist at lower, more relevant doses. These lower doses are particularly important as they better reflect real-life human exposure to BPA and its non-monotonic dose-response [54,55], in which adverse biological and clinical effects may paradoxically be more pronounced, highlighting the need to investigate these concentrations for accurate risk assessment.

In vitro models, such as human-induced pluripotent stem cell (hiPSC)-derived cardiomyocytes [56] and rat H9c2 cardiomyoblasts [14], have provided valuable mechanistic insights into BPA’s cardiotoxic effects, often implicating oxidative stress and epigenetic modifications. However, their translational relevance is limited by the lack of systemic complexity in an in vivo setting. Likewise, zebrafish models have been widely used to study BPA-induced cardiac abnormalities [57,58], but physiological differences between fish and mammals raise concerns about direct extrapolation to human health risks.

Our study aims to overcome these limitations by employing a rodent model with a low-dose exposure paradigm that closely reflects epidemiological BPA levels in humans, revealing significant molecular and structural alterations. Notably, unlike most previous studies, we systematically investigated sex-specific differences in BPA-induced cardiac alterations. BPA is a well-known endocrine disruptor that exerts sex-specific effects primarily through its interaction with estrogen receptors (ERα, ERβ, and GPER) [59]. However, while most findings come from research focused on neurodevelopmental disorders [60,61], knowledge about the sex-specific effects of BPA on the heart remains scarce and limited to a few studies. Our findings contribute to filling this gap, providing important evidence that prenatal BPA exposure disrupts fetal heart development in a sex-dependent manner, primarily through estrogen receptor dysregulation. Given the pivotal role of ERs in cardiac maturation and function [62], such alterations during fetal programming could have long-term consequences on heart physiology and increase susceptibility to CVD later in life. Specifically, BPA exposure led to a significant downregulation of ERα in female fetal hearts while upregulating ERα in males, highlighting a pronounced sex difference. ERα is a key regulator of genomic estrogen signaling, playing a critical role in cardiac development and cardioprotection by modulating gene expression patterns involved in growth, differentiation, and cellular homeostasis [50,51,59]. Its downregulation in females suggests a diminished estrogenic influence during a critical period of cardiac maturation, potentially impairing the programming of key protective pathways and increasing susceptibility to adverse cardiovascular outcomes later in life. Conversely, excessive ERα expression in males may disrupt the balance between genomic and non-genomic signaling, potentially leading to maladaptive responses under stress conditions and altering normal cardiac programming and function. These findings align with previous studies linking ERα dysregulation to cardiovascular disease susceptibility, particularly in response to oxidative and hemodynamic stress [63].

Interestingly, our data also revealed a notable sex-specific difference in GPER expression following BPA exposure, with an upregulation in females and a downregulation in males, mirroring the inverse pattern observed for ERα. This contrasting regulation suggests a distinct yet interconnected mechanism of estrogen signaling disruption. GPER has been implicated in rapid, non-genomic estrogen signaling and plays a crucial role in cardiovascular protection and function [60,64]. The increased GPER expression in females may represent a compensatory response to preserve estrogen-mediated protective effects in the developing heart, despite the loss of ERα-driven genomic signaling. Conversely, the downregulation of GPER in males, coupled with ERα upregulation, suggests a differential regulatory mechanism favoring genomic over non-genomic responses, potentially reducing the overall capacity for estrogen-mediated cardioprotection.

Regarding ERβ, prenatal BPA exposure resulted in a significant reduction in both sexes. ERβ plays a crucial role in maintaining cardiac homeostasis, exerting cardioprotective effects by reducing inflammation, modulating oxidative stress, and mitigating hypertrophic growth [61]. Therefore, its downregulation suggests a loss of these protective mechanisms, potentially promoting a pro-inflammatory and pro-oxidative environment in the developing heart. In line with this hypothesis, we observed a significant increase in NF-κB protein expression in BPA-exposed fetal hearts, with a more pronounced effect in males. NF-κB is a redox- and hormone-sensitive transcription factor that acts as a central mediator of inflammation and cellular stress responses [37]. It is directly modulated by estrogen receptors and can be activated under conditions of oxidative imbalance and endocrine disruption [65]. Its upregulation in our model, particularly in male fetuses characterized by a reduction in ERβ and divergent regulation of ERα and GPER, supports the idea that BPA-induced estrogenic imbalance contributes to the activation of NF-κB–dependent inflammatory pathways during cardiac development.

Consistently, BPA exposure triggered a significant upregulation of pro-inflammatory cytokines IL-1β and TNF-α in males, with a similar but non-significant trend in females. These cytokines are key mediators of cardiac dysfunction [38,66], and their increased expression suggests that BPA exposure primes the fetal heart toward a pro-inflammatory state, potentially exacerbated by the loss of ERβ-mediated regulation. The pronounced inflammatory response in males may be linked to differential compensatory mechanisms involving ERα and GPER, reinforcing the idea of sex-specific vulnerability. The concurrent upregulation of the NLRP3 inflammasome in both sexes further supports the hypothesis that BPA fosters an inflammatory environment in the developing heart. While NLRP3 activation has been linked to adverse cardiac remodeling in adults [67], its precise role in fetal cardiac development remains to be fully elucidated. However, its upregulation suggests a potential mechanism through which BPA-induced inflammation is exacerbated in the fetal heart.

In parallel, BPA exposure impaired antioxidant defenses, as evidenced by the significant downregulation of key myocardial antioxidant enzymes, such as SOD1, SOD2, and CAT, in both sexes [68,69]. This suggests that BPA weakens the heart’s ability to counteract oxidative stress, potentially increasing long-term cardiovascular vulnerability. Furthermore, SELENOT, a crucial regulator of cellular stress response and cardioprotection [26,29,32], was also significantly downregulated in both sexes. Given its crucial role in early development, evinced by studies showing that SELENOT knockout leads to embryonic lethality in mice [70], as well as its involvement in early hyperplastic growth of cardiomyocytes [26], its reduced expression could have lasting consequences on heart physiology and resilience to future cardiovascular challenges. This impaired antioxidant profile was further corroborated by the significant increase in protein oxidation and TBARS levels (a key index of lipid peroxidation [71]) observed in BPA-exposed fetal hearts. This finding indicates that the disruption of enzymatic defenses can translate into measurable oxidative damage within the fetal heart and strengthens the notion that impaired redox homeostasis is a central mechanism through which BPA exerts its cardiotoxic effects on fetal cardiac development.

In light of the observed increase in lipid peroxidation, we reasoned that ferroptosis, a regulated form of cell death driven by iron-dependent lipid ROS accumulation, might be involved in the cardiac effects of prenatal BPA exposure. Supporting this hypothesis, we observed a significant downregulation of GPX4, a key selenoenzyme that detoxifies phospholipid hydroperoxides and inhibits ferroptosis. Conversely, ACSL4, which facilitates the incorporation of polyunsaturated fatty acids into membrane phospholipids and promotes lipid peroxidation susceptibility, was markedly upregulated in BPA-exposed fetal hearts. These alterations were observed in both sexes and are consistent with the elevated TBARS levels, suggesting that BPA-induced redox imbalance may promote ferroptotic signaling as a complementary mechanism of cardiac injury during development. This interpretation is supported by recent evidence showing that BPA may trigger ferroptosis in fetal heart tissue through disruption of the System Xc^−^ and related metabolic regulators, such as SLC7A11 and SLC3A2, ultimately impairing redox homeostasis and cardiac development [72].

Since inflammation and oxidative stress are key drivers of adverse cardiac remodeling, we assessed the gene expression levels of ANP and BNP, well-established markers of myocardial stress that are upregulated in heart failure and myocardial infarction and also used as indicators of ventricular hypertrophy [73,74]. BPA exposure led to a significant upregulation of both, with males exhibiting higher ANP levels than females. This finding suggests that BPA-induced myocardial stress may be more pronounced in males, potentially due to their heightened inflammatory response. Structural changes were further evidenced by the increased activation of MMP2, a key enzyme involved in extracellular matrix (ECM) turnover [75,76], along with elevated gene expression of Col1A1 and Col3A1, markers of fibrotic progression and ECM deposition [44]. These findings indicate that BPA alters ECM homeostasis by disrupting the balance between matrix degradation and excessive collagen accumulation, ultimately contributing to myocardial fibrosis. The upregulation of TGF-β, a central regulator of fibrosis [77], and its downstream effector CTGF [45] further supports the notion that BPA exposure promotes a shift toward a pro-fibrotic cardiac phenotype. Interestingly, the stronger pro-fibrotic response observed in males suggests a sex-specific vulnerability, which may stem from the differential regulation of estrogen-mediated protective mechanisms.

To complement these findings, histological analyses were performed to assess whether the molecular alterations observed translated into structural changes in the architecture of the fetal heart. H.E. staining revealed clear morphological differences between BPA-treated and control groups, particularly in male fetuses. While control hearts exhibited incomplete tissue differentiation, a typical feature of late gestation, BPA-exposed male hearts displayed increased cellularity suggestive of hyperplasia, marked fiber anisotropy, and enhanced vascularization, all indicative of altered cardiac morphogenesis. Additionally, early signs of degenerative processes resembling necrobiosis were observed, suggesting compromised cardiomyocyte integrity. These observations are in line with previous findings by Rasdi et al. (2020) [78], who reported structural disorganization and early myocardial remodeling in fetal rat hearts following prenatal BPA exposure. In contrast, female fetuses displayed only mild structural alterations and a modest increase in vascularization, reinforcing the notion of sex-specific vulnerability.

Masson’s trichrome staining did not reveal overt fibrosis in any group, indicating that, although BPA exposure triggered a clear pro-fibrotic molecular response (e.g., increased TGF-β and CTGF), histological evidence of tissue remodeling was not yet detectable at this developmental stage. This apparent discrepancy may reflect an early and dynamic phase of ECM turnover, in which collagen synthesis is counterbalanced by increased matrix degradation, as suggested by the concomitant upregulation of MMPs. As highlighted by Ghazal et al. (2025) [79], this represents a key transitional state in fibrotic pathogenesis, where molecular activation precedes, and may even predict, structural remodeling that could manifest during postnatal development or in response to later cardiovascular stressors.

PAS staining revealed a reduction in myocardial glycogen content following BPA exposure, with a more pronounced depletion observed in male fetuses. Given the role of glycogen as a critical energy reserve during fetal cardiac development [80], this finding suggests an early impairment in cardiomyocyte metabolic homeostasis. This observation aligns with recent findings by Ermini et al. (2022) [81], who demonstrated that BPA exposure suppresses the expression of GLUT1, the primary glucose transporter in the fetal myocardium, thereby impairing glucose uptake. Notably, the same study also reported an upregulation of the lipid transporter CD36, along with a significant accumulation of triglycerides in BPA-exposed fetal hearts, supporting the hypothesis of an early and maladaptive transition from carbohydrate to lipid utilization. This metabolic shift may represent a compensatory, yet dysfunctional, cellular response to the pro-oxidative and pro-inflammatory environment triggered by BPA exposure [82], as evidenced by the upregulation of inflammatory cytokines and disruption of redox homeostasis in the fetal heart. In further analyzing BPA-dependent structural damage, cardiac immunohistochemical and immunofluorescence results indicated a marked reduction, particularly in male hearts from BPA group, of desmin, a member of the intermediate filaments that provides structural support and mechanical stability to the heart muscle cells, also playing a critical function in cardiac muscle development at the embryonic stage [83]. On the other hand, the analyses showed increased CD34 and Ki67 expression, especially within microvascular structures in female hearts. Interestingly, this is supported by a recent study indicating that BPA-induced vascular alterations appear to be modulated by the interaction of BPS with ERs signaling [84]. These data suggest that prenatal BPA exposure can compromise myocardial fiber architecture and promotes vascular remodeling and proliferative responses in a sex-dependent manner.

Collectively, the histological evidence supports the molecular findings, reinforcing the notion that BPA disrupts multiple aspects of fetal cardiac development. This highlights the particular vulnerability of the developing heart, especially in male fetuses, to endocrine disruption, and underscores the relevance of early life exposure in the programming of long-term cardiovascular risk.

### Limitations

Despite the novel morphological and molecular insights provided by our in vivo findings into the effects of prenatal BPA exposure on fetal heart, some limitations of the study should be acknowledged. We did not provide a functional assessment of the fetal heart vasculature due to considerable technical challenges: the vessels are extremely small and exhibit a dense, highly branched network that, to our knowledge, cannot be reliably cannulated or pressurized to reproduce in vivo hemodynamic conditions. Additionally, while our histological analyses revealed structural alterations in cardiac architecture, they do not allow definitive conclusions regarding the type of cardiomyopathy induced by prenatal BPA exposure. Taken together, these limitations underscore that, from a functional perspective, the cardiovascular implications of prenatal BPA exposure remain incompletely defined. This reflects both the inherent challenges associated with functional assessments in the fetal model, as well as the gaps in integrating morphological alterations with physiological outcomes. Further investigations integrating functional and morphological assessments are warranted to fully elucidate the cardiovascular consequences of BPA exposure in fetal and post-natal conditions.

## 5. Conclusions

Overall, our findings provide novel insights into the developmental origins of cardiovascular disease, emphasizing the urgent need for targeted preventive strategies and further research to elucidate the functional consequences of BPA-induced receptor changes, particularly in relation to postnatal cardiac function and disease susceptibility. Future studies should investigate the precise molecular mechanisms underlying these sex-specific effects, including potential epigenetic modifications and interactions with other hormonal pathways. Additionally, assessing long-term functional outcomes in postnatal and adult offspring will be critical to determining whether fetal alterations translate into persistent cardiovascular dysfunction.

On the other hand, our study underscores the urgent need to reassess BPA safety regulations, highlighting concerns about the adequacy of current exposure thresholds. Existing guidelines primarily address reproductive and metabolic toxicity, often overlooking cardiovascular effects [22]. In this regard, regulatory agencies should consider revising exposure limits, particularly for vulnerable populations, such as pregnant women and developing fetuses, and incorporate developmental cardiac toxicity into comprehensive risk assessments.

## Figures and Tables

**Table 1:** Primer sequences used for qPCR amplification.

Gene Accession Number	Forward Primer 5′-3′	Reverse Primer 5′-3′
NM_031512 (*Il-1β*)	-CCCAGGACATGCTAGGGAGCC-	-AGGCAGGGAGGGAAACACACG-
NM_012675.3 (*Tnf-α*)	-CACCACGCTCTTCTGTCTACTG-	-GCTACGGGCTTGTCACTCG-
NM_012612.2 (*NppA*)	-GGAAGTCAACCCGTCTCAGA-	-TGGGCTCCAATCCTGTCAAT-
NM_031545.1 (*NppB*)	-CCAGAACAATCCACGATGCA-	-GCAGCTTGAACTATGTGCCA-
NM_053304.1 (*Col1A1*)	-GACATGTTCAGCTTTGTGGACCT-	-AGGGACCCTTAGGCCATTGTGTA-
NM_032085.1 (*Col3A1*)	-TTTGGCACAGCAGTCCAATGTA-	-GACAGATCCCGAGTCGCAGA-
NM_021578.2 (*Tgf-β*)	-AACCGACCCTTCCTGCTCCT-	-TCCACTTCCAACCCAGGTCCT-
NR_046237.2 (*18s rRNA*)	-CATTCGAACGTCTGCCCTAT-	-GTTTCTCAGGCTCCCTCTCC-

## Data Availability

Data are contained within the article.

## Abbreviations

The following abbreviations are used in this manuscript:

ACSL4	acyl-CoA synthetase long-chain family member 4
ANP	Atrial Natriuretic Peptide
BNP	Brain Natriuretic Peptide
BPA	Bisphenol A
BPA-GA	BPA-glucuronide
CAT	Catalase
Col1A1	Collagen type I alpha 1 chain
Col3A1	Collagen type III alpha 1 chain
CTGF	Connective tissue growth factor
CVD	Cardiovascular disease
ECM	Extracellular matrix
EDCs	Endocrine-disrupting chemicals
ER	Estrogen receptor
EtOH	Ethanol
GPR30/GPER	G protein-coupled estrogen receptor 30
GPX4	Glutathione peroxidase 4
H.E.	Hematoxylin and eosin
hiPSC	Human-induced pluripotent stem cell
IL-1β	Interleukin-1β
MMPs	Matrix metalloproteinases
NF-κB	Nuclear Factor kappa B
NLRP3	NOD-like receptor protein 3
qPCR	Quantitative real-time PCR
SELENOT	Selenoprotein T
SERM	Selective estrogen receptor modulator
SOD	Superoxide dismutase
TGF-β	Transforming growth factor-β
TNF-α	Tumor necrosis factor-α
